# Industrial Adoption of Carbon Nanotubes

**DOI:** 10.1021/acs.nanolett.5c05572

**Published:** 2026-03-17

**Authors:** Dana Goerzen, Matteo Pasquali, Mijin Kim, Daniel A. Heller

**Affiliations:** † Memorial Sloan Kettering Cancer Center, New York, New York 10065, United States; ‡ 12295 Weill Cornell Medicine, Cornell University, New York, New York 10065, United States; § Department of Chemical and Biomolecular Engineering, Department of Materials Science and NanoEngineering, The Carbon Hub, 3990 Rice University, Houston, Texas 77005, United States; ∥ Department of Chemistry, 1372 Georgia Institute of Technology, Atlanta, Georgia 30332, United States; a Smalley-Curl Institute, Rice University, Houston, Texas 77005, United States

**Keywords:** carbon nanotubes, batteries, composites, materials, sensors, industry

## Abstract

Over the past 30 years, carbon nanotubes have emerged as one of the most exciting classes of nanomaterials due to their unique physicochemical properties. While challenges in nanotube synthesis and processing initially hindered their adoption, many of these barriers have since been addressed by research and manufacturing advances, resulting in substantial industrial application of nanotubes across multiple sectors. However, much of this progress is not known in the academic community. This perspective discusses the current landscape and outlook of industrial integration of carbon nanotubes and key factors mediating widespread integration across all major material-related areas of human activity.

Carbon nanotubes are a heterogeneous class of carbon-based nanomaterials composed of cylindrical layers of graphitic carbon, which can be either a single layer, called single-walled carbon nanotubes, or several-to-many concentric tubes, collectively called multiwalled carbon nanotubes. Carbon nanotubes have been of interest to the nanomaterials research community since their synthesis and (re)­discovery in 1991,^1^ due to their unique physicochemical characteristics. At the nanoscale, they can be stronger than steel but have a lower density;^2^ be more conductive than copper^3^ or be single-molecule semiconductors;^4^ or exhibit a higher thermal conductivity than diamond.^5^ However, the realization of these exciting properties as a bulk material did not materialize rapidly, prompting some degree of confusion and disillusionment in the broader community.

Over the past 30 years, academic and industrial researchers have made significant progress in addressing issues relating to carbon nanotube purity,^6^ scalability,^7^ and post-synthetic processing.^8^ Some applications have reached full industrial adoption and technological maturity. However, a significant knowledge gap exists between the research and industry communities regarding the state and needs of industrial applications of carbon nanotubes and the outstanding questions to be collaboratively addressed. In this perspective, we outline recent developments in the industrial adoption of carbon nanotube-based materials, emphasizing applications currently at commercialization and investment stages. We also provide an outlook on up-and-coming applications, investments, and challenges that must be addressed for carbon nanotubes to be integrated into many areas of industrial activity.

## Private Sector Adoption of Carbon Nanotube Materials

Advances in carbon nanotube synthetic processes have begun to support industrial applications at scale and at increasingly low costs. Sustained research efforts into processing and novel applications have resulted in a mature body of knowledge in many sectors, facilitating the translation of laboratory findings to real-world applications, ranging from highly developed applications such as battery components to the preliminary application of biomedical diagnostics and chemical sensing.^9^ As a result of these advances, venture capital, and internal corporate investments are supporting carbon nanotube-related businesses in a wide range of fields.

### Developments in Carbon Nanotube Manufacturing, Synthesis, and Purification

Carbon nanotube manufacturing is driven by sectors such as electronics, energetics, and textiles.^9,86^ *LG Chem*, a major producer of carbon nanotube materials for electronics and energy storage (batteries) applications, projects that 2030 production will exceed 95,000 tons.^10^ Various applications require different carbon nanotube properties and production volumes to achieve economically viable applications. Currently, multiwalled carbon nanotubes dominate production due to simpler, cost-effective synthesis. In energy storage applications, at-scale synthetic methods to selectively produce multiwalled carbon nanotubes are well-established for conductive additives of lithium-ion batteries and have significant potential for rapid industrialization. Similarly, these multiwalled nanotubes can be used in polymer nanocomposites, primarily for antistatic dissipation. Conversely, high-aspect ratio, crystalline, few-walled nanotubes are required for making strong fibers and other macroscale materials^11,12^ Semiconductors and high-performance transistor applications of carbon nanotubes require ultrapure semiconducting single-walled carbon nanotubes.^13^ Some high-value applications, such as aerospace engineering and biomedical applications, need relatively little carbon nanotube material but require high physicochemical integrity and specific formulations of nanotube material. The high-quality of carbon nanotubes in these cases, can be equally or more important than production volume and cost reduction.

There have been consistent increases in production to meet the escalating demands of these sectors, with extensive investment in manufacturing plants.^10^ Over the past decade, there has been an upward trajectory of private investment in carbon nanotube companies that produce diverse types of carbon nanotube materials. For example, OCSiAl and CHASM Advanced Materials Inc. produce various forms of single-walled and multiwalled carbon nanotubes that can be readily adapted for diverse applications, including plastics, aerospace, and batteries. Batteries are also the largest application market of Meijo Nano Carbon. Nantero focuses on carbon nanotube-based memory, and Mitsui Chemicals commercializes carbon nanotube-based UV-lithography pellicles. Despite the fact that, in many of these cases, capacity growth and price decreases have been limited because of the difficulty to secure funding for plant construction, steady increases in nanotube production have broadened the applications in which they are economically viable and are attributable to refinements in production methodologies (including catalyst design and process optimization), alongside the adoption of sustainable feedstock sources.

Depending on the applications and demands, different synthetic and processing techniques are optimized for industrialization.^9^ Chemical vapor deposition (CVD) is a popular method for producing carbon nanotubes, especially valued for its precise control over nanotube characteristics like length, diameter, alignment, and purity at large scale.^14^ In floating-catalyst CVD, the main issue remains reactor intensification, where the recent advances from Huntsman^15^ and OCSiAl^16^ appear most promising. In supported catalyst CVD, the removal of carbon nanotubes from the catalyst support remains problematic, although there have been promising advances^17,18^ and, in some cases such as cement additives, the catalyst support can be left in the product.^19^ Laser ablation is known for producing single-walled carbon nanotubes with high quality and low metallic impurities, but this method is not economically advantageous for large-scale production, and nanotube alignment is difficult to control.^20^ Carbon arc discharge is effective for creating fewer structural defects in single-walled and multiwalled carbon nanotubes and is scalable, but it offers little control over nanotube alignment and requires purification due to metallic catalysts.^20^

Recent developments in nanotube synthesis and purification may facilitate homogeneous productions,^6^ for specialized applications. Novel synthetic methods using innovative catalyst designs^21^ or catalyst-free methods using purified carbon nanotubes as “seeds”^22^ may address limitations in homogeneous nanotube synthesis, although their industrial translation is still limited to processes like CoMoCAT, which is selective toward a single chirality of single-walled carbon nanotubes.^23^ However, ultrapure synthesis of monochiral carbon nanotubes remains relatively low-yield, and few different carbon nanotube chiralities can be synthesized selectively.^6^ The purification of carbon nanotubes involves various steps, e.g., partial thermal oxidation^24^ and acid treatment,^25^ to remove impurities like amorphous carbon, metal catalyst particles, and other carbonaceous materials. Filtration and centrifugation steps can separate carbon nanotubes from larger graphite particles and solvents.^26^ These methods vary in complexity and effectiveness and are crucial for achieving the high purity required for some applications.

Despite remaining challenges in scaling up synthesis and purification of carbon nanotube materials, carbon nanotubes dominate in industrial applications compared with other nanotube materials, such as boron nitride nanotubes and transition metal dichalcogenide (TMD) nanotubes. While boron nitride nanotubes share similar mechanical and thermal dispersion properties, their production remains limited to the scale of grams-per-day due to difficulties in large-scale synthesis.^27^ In contrast, carbon nanotubes can be mass-produced at the ton scale, facilitating their widespread integration into lithium-ion batteries, structural composites, and high-value thermal dispersion and biomedical applications. Consequently, industrial uses of boron nitride nanotubes are currently limited to specialized high-temperature or radiation-shielding applications. Producing and purifying structurally well-defined TMD nanotubes is still in the early stages in research laboratories due to the strain energy associated with rolling up the three-atom-thick TMD monolayers.^28^ Despite differences in physicochemical properties, researchers account for an analogy with carbon nanotubes to understand the structure–property relationships of these emerging nanotube materials and to achieve scalable synthesis, purification, characterization, and commercialization.

Carbon nanotube technology has transitioned into high-volume supply chains, with annual global production capacity now exceeding 5000 tons. Current deployments meet the technical rigor required for modern industrial standards (Table 1). In the energy sector, companies like LG Chem and Jiangsu Cnano have integrated multiwalled carbon nanotubes as standard conductive additives, achieving up to a 20% reduction in internal resistance in EV battery cathodes. In the electronics supply chain, Mitsui Chemicals has operationalized the mass production of carbon nanotube-based EUV pellicles, maintaining >94% transmittance while withstanding high-power (>600W) lithography environments.

**Table 1:** Representative Commercially Available CNT-Enabled Products

Company	CNT type	Product form	Target specifications/performance	Application sector	Reference (company site, review, or trade source)
NanoIntegris	SWCNT	Powder, surfactant solution	95–99% purity; separated metallic vs semiconducting (>99% chirality).	Electronics: Thin-film transistors, sensors	^29^
Hyperion Catalysis	MWCNT	FIBRIL (Concentrate)	Conductivity: Percolation reached at 2–5 wt %; High aspect ratio of 1000:1 maintains resin ductility	Electronics/Auto: Electrostatically dissipative fuel lines and IC trays	^30^
Nano-C	SWCNT	Powder/Inks	Chirality control: High-purity single-walled carbon nanotubes tailored for solution processing.	Electronics: Transparent conductive films, sensors, and photodetectors.	^31^
Toyocolor	MWCNT	LIOACCUM (Dispersion)	High-dispersion conductive additive; replaces carbon black to reduce resistance.	Energy: Li-ion batteries for Toyota HEVs.	^32^
Jiangsu Cnano Technology	MWCNT	LB Series (Dispersions)	3%–10% carbon nanotube loading reduces internal resistance by ∼ 20% compared to carbon black; 15%–20%carbon nanotubes (FloTube) to achieve 2–5 wt % percolation	Energy: EV battery supply chain	^33^
		Masterbatch (Pellets)		Electronics/Auto: Electrostatic discharge protection	
DexMat	MWCNT	Galvorn (Fibers, Yarns, Tapes)	Density: 1.6 g/cm^3^ (80% lighter than Cu); Conductivity: up to 10 MS/m.	Aerospace/Data: EMI shielding, lightweight signal wiring	^34^
LG Chem	MWCNT	Powder, paste	Conductivity: 10% higher than carbon black; Loading: Reduces additive volume by 30% to increase active material density.	Energy: Conductive additives for high-nickel cathodes in EV batteries	^35^
				Materials: engineering plastic used for electrostatic powder coating	
Meijo Nano Carbon	SWCNT	MEIJO eDIPS (Ink/Yarn)	High crystallinity; available as high-purity semiconductor-only ink	Electronics: Printed electronics, flexible semiconductor devices	^36^
Nantero	MWCNT	NRAM (Semiconductor Memory)	High-speed, nonvolatile memory; high heat/radiation resistance.	Electronics: Enterprise storage, automotive computers	^37^
Mitsui Chemicals	MWCNT	Pellicle (Thin Film)	EUV transmittance: ≥ 94%; Heat Resistance: Withstands >1 kW exposure power	Electronics: Next-gen EUV semiconductor manufacturing.	^38^
OCSiAl	SWCNT	TUBALL (Additive/Masterbatch)	>90% G/D ratio; effective at <0.1% loading; preserves transparency/color.	Electronics/Auto: Antistatic coatings, Li-ion battery electrodes.	^39^
CHASM Advanced Materials Inc.	SWCNT	Signis (VC102 Ink)	Stability: Passed 1,000h at 65 °C/85% RH; replaces PEDOT in harsh environments.	Electronics: Transparent flexible touch buttons and wearable devices.	^40^
	SWCNT/Hybrid	AgeNT (Transparent conductive film)	Sheet Resistance: < 10 Ω/sq at >90% transparency; flexible/thermoformable	Electronics/Auto: ADAS heaters, transparent 5G antennas, and touch sensors	^40^
	MWCNT/SWCNT	NTeC-E (Conductive Additive)	High conductivity and capacity retention	Energy: Conductive additives for Li-ion cathodes and silicon anodes in EV batteries	^40^
Huntsman	MWCNT	MIRALON (Sheets, Yarns, Dispersions)	25x stronger than steel; used as current collectors to replace Al/Cu foils.	Space/Energy: NASA Juno (ESD protection), Li–S batteries.	^41^

### Trends in Carbon Nanotube-Related Intellectual Property and Investment

To assess the industrial activity of carbon nanotube materials, we conducted a textual analysis of 339,157 patent applications and 107,404 patent approvals in the United States of America Patent Trade Office database from 1994 to 2023 (Figure 1). Textual analysis on the database indicates that annual patent applications have increased linearly over the past 29 years, with over 20,000 patent applications filed in 2023 (Figure 1A), suggesting robust interest in the commercialization of carbon nanotube applications—attributable, in part, to advances in scalable carbon nanotube synthetic methods, processing techniques, and novel applications. The strong private sector interest in industrial applications of nanotubes is reflected in patent ownership, with private companies holding 84.7% of approved patents, compared to 15.3% by research institutes (Figure 1B inset).

**Figure 1:**
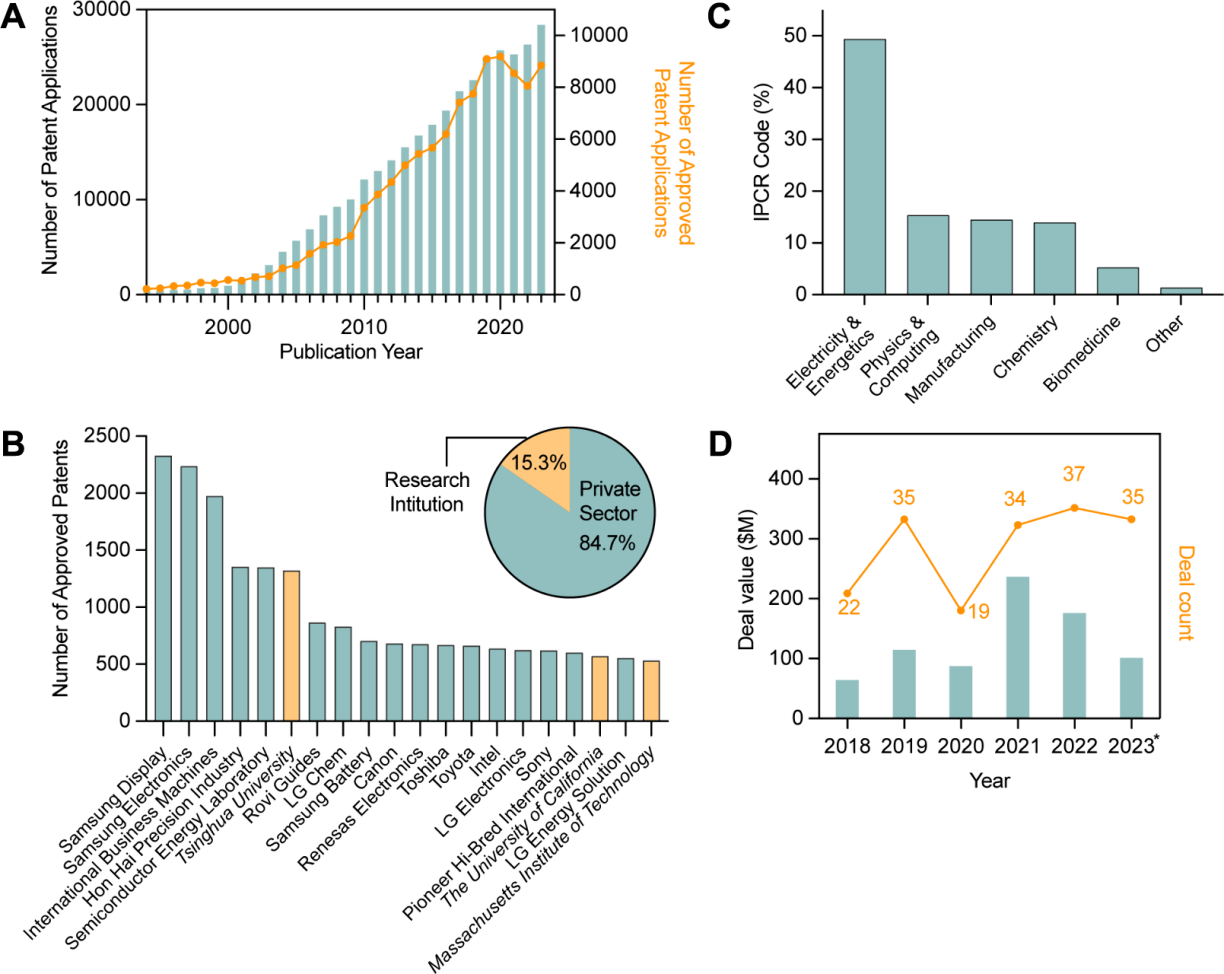
Textual analysis of patent and industrial application breakdown from 1994 to 2023. (A) Annual number of global carbon nanotube patent applications, from 1994 to 2023. (B) List of patent owners with over 500 patents relating to carbon nanotubes. Inset pie chart: Proportion of patents held by private sector and research institutions. (C) Distribution of Reformed International Patent Classification (IPCR) codes by major classes of claim. (D) Global venture capital activity of carbon nanotubes. Data from the year 2023 is up to date as of January 9, 2024. Source: PitchBook Data. Inc.

Carbon nanotube intellectual property is primarily held by companies focused on electronics and energy storage, e.g., Samsung Electronics and IBM (Figure 1B). Many of these patents are related to LEDs, batteries, and semiconductors. Reformed International Patent Classification (IPCR) analysis of codes assigned to carbon-nanotube-related patents shows that approximately 50% of patents relate to energy storage, while computing-related, manufacturing-related, and chemistry-related patents comprise 16% each (Figure 1C). Importantly, many of the foundational patents on composition of matter, synthesis, dispersibility, etc., have now expired.

Early industrial applications typically involved carbon nanotube composites comprised of mixtures of unaligned, heterogeneous nanotube materials, often to enhance the mechanical strength and flexibility of commercial materials. As a result, early applications often did not harness the exceptional mechanical and electrical properties observed at the nanoscale. Recent advances in nanotube synthesis and processing have translated to the macroscale their exceptional nanoscale mechanical and electrical properties.^42^ Many of these innovations are now approaching commercialization, driving investment in carbon nanotube startups and the broader advanced materials sector.

Carbon nanotube applications are currently attracting substantial financial investment, according to PitchBook Data, Inc. (Figure 1D). In 2023, 35 new investment deals collectively worth $100 million were completed.^43^ Most investment capital is focused on companies developing nanotube-enhanced batteries or other application-driven ventures. Manufacturing companies synthesizing highly pure carbon nanotube stocks at scale are primarily funded by internal corporate investments rather than venture capital. As of January 2024, the National Science Foundation and US Department of Defense were among the largest governmental funding agencies for carbon nanotube startups, with 180 Degree Capital, and Charles River Ventures leading private sector investments into nanotube deals.^43^

## Focus Areas of Carbon Nanotube Research

There have been sustained and significant research efforts into innovative carbon nanotube-based materials that can be applicable to commercial manufacturing facilities and applications. Broadly, three main groups of carbon nanotube research activities relevant to industry include scalable production of carbon nanotubes, translation of fundamental research findings to commercial products, and efforts to address sustainability questions.

As-produced carbon nanotubes are often heterogeneous in length, diameter, wall numbers, *etc.*, and synthesis/postprocessing needs vary according to application. New synthetic and processing methods^44,45^ have significantly improved the diverse aspects of carbon nanotube production and quality control to meet the market needs. To translate unique carbon nanotube properties at the nanoscale to macroscale composite materials, physicochemical characteristics of carbon nanotubes, including length, defect density, alignment, *etc.*, have been extensively interrogated for each application. Improvements in scalability of chirality-controlled synthesis, separation, and purification methods, as well as the creation of nanocomposite materials in scale, are of substantial interest.

The development of novel uses, form factors, and composite arrangements is an active research area. High-performance carbon nanotubes have been developed into various structures, e.g., fiber, yarn, film, fabric, and composite materials.^46^ By employing techniques such as electric field-assisted alignment and template-directed assembly,^47^ researchers have achieved precise control over the orientation of carbon nanotubes within a matrix. Once carbon nanotubes have been processed into different intermolecular arrangements, they can be used for a wider variety of applications appropriate to their new forms.^48,49^ Highly packed, well-aligned carbon nanotubes translate to the macroscale a meaningful (∼10–20%) fraction of the tensile strength and stiffness of individual nanotubes^42^ and can be demonstrated at a small industrial scale.^46,50^ Interestingly, when used in composites (e.g., pressurized gas storage tanks), carbon nanotube fibers outperform commercial carbon fibers, possibly because of better interfacial stress transfer with the matrix.^51^ Another benefit of alignment and packing density is improved electrical and thermal conductivity, in addition to their high tensile strength, making them ideal candidates for structural and thermal dispersive applications in aerospace and automotive, e.g., thermoelectric applications.^52^ The availability of these fibers to the research community has shown emergent properties that were not initially anticipated. In the biomedical area, the discovery of low interfacial impedance upon contact with skin or biological tissues has enabled research-stage applications in wearables,^53^ electrophysiology,^54^ and neuroengineering.^55^ In power electronics, the finding of combined low skin effect and low proximity effect has led to the use of carbon nanotube fibers in wireless chargers.^56,57^ In addition, on a much smaller scale, the tailored patterning of nanotubes on transistors and semiconductors will facilitate industrial advancements in carbon nanotube-based electronic components.^13,58,59^

The development of highly purified carbon nanotube materials has facilitated biomedical applications, some of which are beginning to enter industrial translation. Functionalized carbon nanotubes have emerged as versatile platforms for drug delivery,^60^ imaging,^61^ sensing,^62^ and tissue engineering.^63^ By modifying the surface chemistry of carbon nanotubes with biomolecules or targeting ligands, biocompatible nanotubes can be generated, resulting in targeted cellular uptake, paving the way for targeted drug delivery systems^64^ and theragnostic platforms.^65^ The development of carbon nanotube-based scaffolds and hydrogels has shown promise in promoting cell adhesion, proliferation, and differentiation for tissue regeneration,^66^ and in structural composites to mimic bone.^67^ Carbon nanotube-based molecular sensing relies on the unique physicochemical properties of carbon nanotubes to detect analytes or cellular/disease processes through electronic or optical transduction. A promising avenue of carbon nanotube research includes the development of nanotube sensors to improve drug screening,^68^ health monitoring,^69^ and diagnostics.^62,87^

## Promoting Adoption of Carbon Nanotube Materials Across industries

The speed and feasibility of deployment of carbon nanotube materials will largely depend on the following factors (Figure 2):

**Figure 2:**
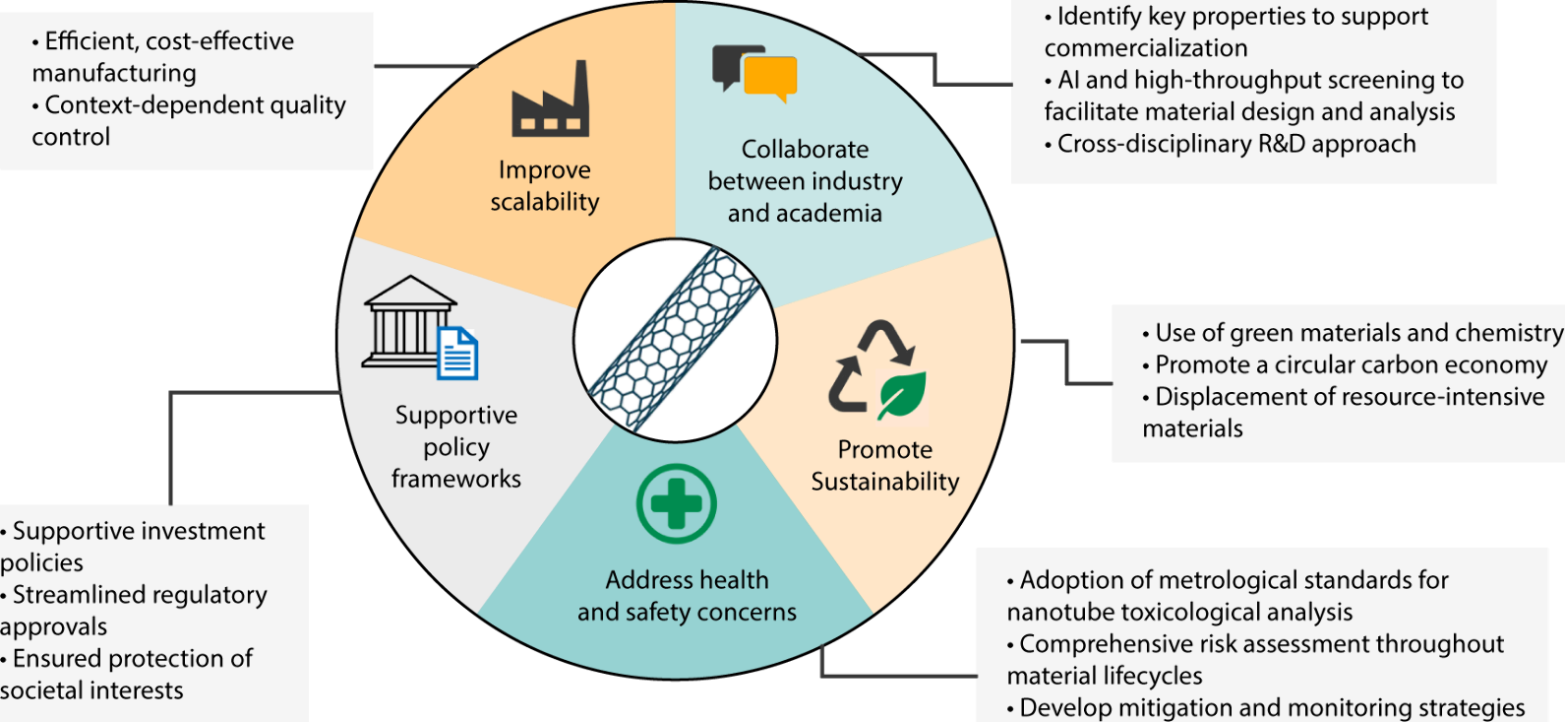
Key considerations to promote industrial adoption and market growth of carbon nanotube applications.

### Scalability

The successful translation of carbon nanotubes from research material to industrial applications hinges on the scalability of manufacturing processes to meet the growing demand for carbon nanotube-based products across a range of industries.^9^ Here, the versatility and unique combination of properties of carbon nanotubes can complicate the efforts of manufacturers to build price-volume curves, which are crucial for developing and deploying economically viable processes. This challenge arises as the same nanotube materials can be used in applications with disparate costs and volume requirements. To balance quality and specifications, industrial requirements of carbon nanotube formulations should be identified for each application, with scalability and quality control processes tailored accordingly. For example, while aerospace applications require extremely high-quality carbon nanotubes with minimal sidewall defects, energy storage applications can tolerate higher defect densities depending on specific requirements. While conventional materials like carbon black and graphene offer lower upfront costs, the economic feasibility of carbon nanotube materials is driven by value-in-use at low loading levels. Carbon nanotubes offer a unique value proposition in energy and electronics, where their performance often offsets the high cost of materials in the global high-tech supply chain (Table 1).

### True Collaboration between Academia and Industry

Steady advances in carbon nanotube research over the past three decades have improved the fundamental understanding of their physicochemical properties. However, progress in refining their properties, and in particular their logistical and economical requirements such as scalability and cost, is advancing at different rates across applications, resulting in uneven commercial adoption. Translation beyond academic research and into the production of carbon nanotube materials with economically valuable properties at scale may be facilitated by several areas of improved collaboration. Collaboration should not be just between academic researchers and commercial producers (e.g., collaborations between Tortech and Cambridge University; CNano and Tsinghua University; Meijo Nano Carbon and Zeon Nanotechnology with AIST, Tsukuba, and other Japanese institutions), but should be extended to other areas of a fledgling carbon nanotube supply chain, including academic researchers working on downstream nanotube applications, and companies that have not traditionally been in the carbon nanotube space but have the appropriate technological and commercial toolsets to bring new products to market. A promising example is the acquisition of NanoComp by Huntsman Corp. NanoComp was one of the earliest startups in the production of carbon nanotube fibers via direct spinning from floating catalyst CVD reactors; over about 15 years, the company made impressive technological advances and developed novel fibers with valuable properties but was unable to attain production costs that would have allowed significant market adoption. As an established manufacturer of advanced materials, Huntsman has made significant progress in cost reduction, recently announcing a new 30-ton production facility under construction and is working with industrial and academic partners on applications that could provide uptake at the projected production and cost levels.^70^

There are opportunities for private-public investment in collaboration between national funding agencies, academia, and industry that provide the contractual frameworks that enable multiparty collaborations (e.g., NSF Engineering Research Centers and Science & Technology Centers). Much of the research needed to develop scalable processes at economically favorable prices cannot be undertaken by current industry, which is mostly comprised of startups, due to high capital requirements and mismatched time scales (startups can rarely afford multiyear efforts to develop new processes). Within the framework of collaborative research centers, the likelihood of successful technology transfer from bench to market would increase as academic and industrial researchers work across a value chain. Workforce exchange programs would be facilitated by the existence of such centers, e.g., doctoral or postdoctoral internships, coadvising of doctoral and postdoctoral researchers by industrial scientists, or academic-industrial sabbaticals.

Active discussions between industry, academia, regulators, and funding organizations will help collaboratively address technological, sustainability, and regulatory barriers. However, the process of disseminating academic findings to industrial applications is inefficient. Academic findings reported through scholarly journals and scientific conferences are often naïve about industrial challenges and may be discounted altogether—an example is the use of argon in many carbon nanotube synthesis academic studies, which is done for convenience yet often puts off industrial researchers evaluating processes for commercial applications. The development of industry-focused platforms for sharing research could help bridge the communication gap. Meetings explicitly designed to bridge between industrial stakeholders and academics, such as the Carbon Hub Annual Meeting,^71^ are a key component of such knowledge transfer, and continued investment and expansion of such forums is merited. Additionally, improving cross-pollination between academic and industry-focused conferences to include a broader set of participants could benefit both parties, such as the more academic-focused International Conference of the Science and Application of Nanotubes and Low-Dimensional Materials and the Nanocarbons Division symposia at The Electrochemical Society meetings.

### Sustainability

Carbon nanotubes offer the potential to address climate challenges and support a sustainable carbon economy.^72^ Yet, this will require reactor intensification and scale-up of current processes. Additional opportunities to improve energy intensity in carbon nanotube synthesis include recycling carbon feedstock in current synthetic methods and coproducing hydrogen wherever hydrocarbons are used as feedstock.^73^ Due to their physical stability, carbon nanotubes appear to survive recycling processes without substantial damage.^74^ The development of efficient separation and recycling solutions for carbon nanotube products would significantly extend their useful lifetime.^72^ Where carbon nanotubes and/or their composites displace energy-intensive and heavy materials, the applications also benefit from being lightweight to improve fuel and energy efficiency in transportation.

Various feedstocks in addition to “classical” ones (hydrocarbons, alcohols) are being investigated, including biochar, biomass, and even CO_2_.^75,76^ Although, on the surface, these alternate feedstocks may appear attractive because of their perceived environmental friendliness, a full prospective life-cycle analysis (with realistic assumptions on the electrical energy mix) will need to be conducted to assess which of these feedstocks may be most sustainable.

Another positive environmental impact of the adoption of carbon nanotube materials is their ability to displace resource-intensive materials such as steel, aluminum, copper, and concrete, thus reducing environmentally destructive mining and intense chemical processing.^77^ For example, cement production accounts for 8% of global CO_2_ production.^78^ The addition of carbon nanotubes (0.1 wt %) significantly improves the mechanical properties of cement and increases its physical robustness to cracking.^79^ The introduction of 5 wt % carbon nanotubes in steel can improve its hardness by over 40%, significantly reducing the amount of steel needed for a given application.^80^ Where carbon nanotubes or their composites displace energy-intensive and heavy materials, the applications also benefit from lightweight for transportation and more efficient use of fuel and energy

### Addressing Human Health and Environmental Safety Concerns

Conflicting toxicology studies and a lack of standardization in toxicological and safety assessments have diminished the public perception of carbon nanotubes and have resulted in calls for increased regulatory oversight in the US and the EU (Table 2). Addressing safety concerns of nanotube materials throughout their life cycle^27^ is critical to ensure that carbon nanotubes are being deployed in a safe manner and that any potential negative effects are adequately controlled. Nomenclature and standards that were developed almost two decades ago need to be refined to reflect the differentiation of grades and uses that has occurred in practice. Continued research efforts should be directed toward comprehensively understanding the potential risks associated with carbon nanotube exposure in a variety of form-factors,^27^ and in developing effective mitigation strategies to minimize adverse effects on human health and the environment. Establishing robust risk-assessment frameworks and regulatory guidelines will be essential to ensure the responsible development and deployment of carbon nanotube technology, guiding manufacturers and stakeholders in adhering to safety standards and best practices. Additionally, ongoing monitoring and surveillance will be necessary to track any emerging risks or environmental impacts associated with carbon nanotube use, enabling timely interventions and continuous improvement in safety measures.^27^

**Table 2:** Brief Description of Regulations Pertaining to Carbon Nanotube Adoption in industry

Policy	Agency	Description	Date	Region
EU-REACH (Registration, Evaluation, Authorisation and Restriction of Chemicals Act)^81^	European Chemical Agency (ECHA)	EU-REACH regulation requires thorough registration, evaluation, and authorization processes to ensure the safe use of carbon nanotubes. It imposes strict reporting and evaluation practices for manufacturers and importers of carbon nanotubes and exposure reporting requirements for uses of carbon nanotubes.	2007	EU
NIOSH Guidelines on Occupational Exposure to Carbon Nanotubes and Nanofibers^82^	Centre for Disease Control and Prevention (CDC)	Guidelines for employers to ensure safe limits of exposure to carbon nanotubes include:	2013	USA
		Using available information to continually assess current hazard potential related to carbon nanotube exposures in the workplace and protect worker health.		
		Identify and characterize processes and job tasks where workers encounter bulk (“free form”) nanotubes or nanotube composites.		
		Substitute, when possible, a nonhazardous or less hazardous material for carbon nanotubes. When substitution is not possible, use engineering controls as the primary method for minimizing worker exposure.		
		Establish criteria and procedures for selecting, installing, and evaluating the performance of engineering controls to ensure proper operating conditions.		
		Routinely evaluate airborne exposures to ensure that worker exposures are being maintained below the NIOSH recommended exposure limit of 1 μg/m^3^.		
TSCA Section 4: Final Reporting Rule on Nanoscale Materials^83^	Environmental Protection Agency (EPA)	This rule requires manufacturers and importers of carbon nanotubes to notify the EPA of the following information: Chemical identity, method of manufacture, volume of production, processing, use, and exposure information, and health and safety data	2017	USA
ISO/TC 229, ISO/TS 80004, and ISO/TR 13014 ^84^	International Standards Organization (ISO)	The ISO technical committee responsible for nanotechnologies (ISO/TC 229) focuses on nanotechnology standardization, including terminology, measurement, characterization, health, safety, and environmental considerations.	2005	International network of 11 national standards bodies
		ISO/TC 229 published a technical series ISO/TS 80004 which provides standardized terminologies for engineered nanomaterials, including carbon nanotubes.		
		ISO/TC 229 also directed a technical report ISO/TR 13014 regarding guidelines to assess the toxicological impact of engineered nanomaterials, including carbon nanotubes.		
Substitute It Now (S.I.N.) list^85^	ChemSec	The S.I.N. list is intended to promote substitution of highlighted chemicals prior to EU-REACH legislation.	2019	Europe
		Carbon nanotubes were added to the S.I.N. list due to concerns about persistence, reproductive harm, and carcinogenicity.		
TSCA Section 4: Significant New Use Rule (SNUR) on multiwall carbon nanotubes	Environmental Protection Agency (EPA)	Under SNUR, 4 types of multiwalled carbon nanotubes (listed below) need to be preregistered with the EPA 90 days prior to import, manufacturing, or usage in downstream products.	2023	USA
		Multiwalled carbon nanotubes; 4.4–12.8 nm diameter; bundle length 10.6–211.1 μm		
		Multiwalled carbon nanotubes; 5.1–11.6 nm diameter; bundle length 1.9–552.0 μm		
		Multiwalled carbon nanotubes; 7.9–14.2 nm diameter; bundle length 9.4–106.4 μm		
		Multiwalled carbon nanotubes; 17.0–34.7 nm diameter; globular shape		
Creating Helpful Incentives to Produce Semiconductors for America Act (CHIPS Act)	National Institute of Standards and Technology (NIST)	The CHIPs Act supports the advancement of cutting-edge semiconductor technologies, including carbon nanotube-based system-on-chips, by facilitating research and development, enhancing production capabilities, and fostering innovation through collaborations between government entities, industry leaders, and educational institutions. It provides ∼$52B for domestic manufacturing and scientific innovation in semiconductor production.	2022	USA

### Policy Considerations

The implementation of effective policy will play a key role in shaping the trajectory of carbon nanotube technologies from research laboratories to industrial applications. Clear and supportive policies that promote investment in carbon nanotube applications, streamline approval of nanotube manufacturing and usage, and demonstrate effective assessment and protection of societal interests, will foster market growth and scientific advances in carbon nanotube technology (Table 2). By prioritizing investments in early to-midstage R&D, policymakers can stimulate innovation and commercialization within the carbon nanotube industry, which will help address outstanding challenges in synthesis purity and scalability that have thus far hindered industrial adoption. Clear and streamlined approval processes established by regulatory agencies will facilitate technological progress while ensuring that safety and efficacy standards are met, providing confidence to investors and stakeholders in the reliability of carbon nanotube-based products.

Carbon nanotubes have emerged as a promising class of nanomaterials with significant potential in multiple industrial sectors. Over the past three decades, extensive research efforts have identified their exceptional physicochemical properties and addressed limitations in purity and manufacturing scale, resulting in practical applications in electronics, energy storage, aerospace, biomedical technologies, and environmental remediation. Industrial adoption of carbon nanotubes has been facilitated by scientific advancements, paving the way for substantial manufacturing outputs. Private sector investments, notably in sectors such as energy storage and high-performance computing, underscore the growing confidence in carbon nanotubes as a pivotal component of future technologies. Several barriers to the broad industrial adoption of carbon nanotubes remain, including enhancing scalability, ensuring principles of sustainability are considered, and comprehensively addressing health and environmental safety concerns throughout their life cycle. Enhanced collaboration between academia, industry, and regulatory bodies would accelerate innovation, promote wider adoption, and ensure responsible deployment of carbon nanotube technologies. In addition, the development of supportive policy frameworks will play a crucial role in shaping the trajectory of carbon nanotubes from laboratory discoveries to industrial-scale applications.

## Supplementary Material



## Notes

Underlying patent data used for Figure 1. Each worksheet corresponds to a specific panel in the figure: annual counts of U.S. patent applications (1994–2023), patent approvals for the top 20 patent owners over the same period, institutional distribution (research vs commercial) among the top 1,000 patent holders, and summary counts by IPC classification code for granted patents (1994–2023). (XLSX)
